# EMBOLDEN: CATALYZING RESEARCH FOR OLDER ADULTS THROUGH CO-DESIGNED MOBILITY-PROMOTING INTERVENTION RESEARCH

**DOI:** 10.1093/geroni/igad104.1964

**Published:** 2023-12-21

**Authors:** Rebecca Ganann, Caroline Moore, Stuart Phillips, Elizabeth Alvarez, Bruce Newbold, Sarah Neil-Sztramko, Maggie MacNeil, Kathryn Fisher

**Affiliations:** McMaster University, Hamilton, Ontario, Canada; McMaster University, Hamilton, Ontario, Canada; McMaster University, Hamilton, Ontario, Canada; McMaster University, Hamilton, Ontario, Canada; McMaster University, Hamilton, Ontario, Canada; McMaster University, Hamilton, Ontario, Canada; McMaster University, Hamilton, Ontario, Canada; McMaster University, Hamilton, Ontario, Canada

## Abstract

Mobility and social participation are requisites for optimizing health, independence, and quality of life in older populations. Most mobility-enhancing interventions for older adults have been designed by researchers alone, which often experience implementation challenges and limited effectiveness when translated to real-world contexts. The EMBOLDEN research program aims to i) partner with older adults and service providers to co-design, implement, and evaluate a mobility-promoting intervention and ii) enhance physical and mental health, foster social connections, and support system navigation among older adults living in neighbourhoods with health inequities. EMBOLDEN has completed two phases: (1) intervention co-design based on evidence reviews, environmental scan, qualitative study, and strategic guiding council expertise; (2) a mixed-methods pilot randomized controlled trial. Twenty-seven local older adults (≥55y) and 34 service providers contributed to Phase 1, co-designing an integrated health and social care intervention delivered through collaboration with primary care, public health, and recreation. The three-month community-based program includes a weekly interactive group program of physical activity, healthy eating, socialization, and individual system navigation support. The co-design determined priority intervention features (e.g., addressing social determinants of health, assets-based approaches) and implementation strategies. The pilot study evaluated quantitative and qualitative participant outcomes and experiences and intervention team experiences. The pilot study demonstrated intervention feasibility and acceptability to participants and interventionists, informing a larger pragmatic hybrid implementation-effectiveness trial that is currently underway. EMBOLDEN’s partnership engagement was critical to successfully co-designing intervention research to address identified health, mobility, and social needs of older adults living in neighbourhoods with significant health inequities.

